# Role of MicroRNAs in Diagnosis, Prognosis, and Treatment of Acute Heart Failure: Ambassadors from Intracellular Zone

**DOI:** 10.31661/gmj.v9i0.1818

**Published:** 2020-06-23

**Authors:** Seyyed-Reza Sadat-Ebrahimi, Naser Aslanabadi

**Affiliations:** ^1^Cardiovascular Research Center, Madani Heart Center, Tabriz University of Medical Sciences, Tabriz, Iran

**Keywords:** Heart Failure, MicroRNA, Diagnosis, Prognosis, Treatment

## Abstract

Acute heart failure (AHF) is one of the burdensome diseases affecting a considerable proportion of the population. Recently, it has been demonstrated that micro-ribonucleic acids (miRNAs) can exert diagnostic, prognostic, and therapeutic roles in a variety of conditions including AHF. These molecules play essential roles in HF-related pathophysiology, particularly, cardiac fibrosis, and hypertrophy. Some miRNAs namely miRNA-423-5p are reported to have both diagnostic and prognostic capabilities. However, some studies suggest that combination of biomarkers is a much better way to achieve the highest accuracy such as the combination of miRNAs and N-terminal pro b-type Natriuretic Peptide (NT pro-BNP). Therefore, this review discusses different views towards various roles of miRNAs in AHF.

## Introduction

**Figure 1:**
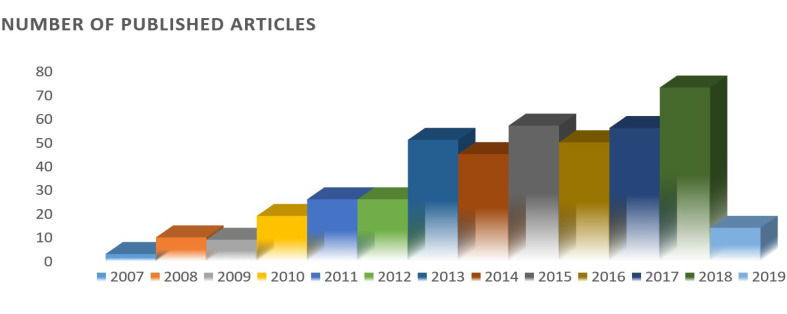
Number of medline-indexed publications per year obtained from www.Pubmed.com at 12/8/2019 using the keywords of (“Heart Failure”[Mesh]) and “MicroRNAs”[Mesh]

Acute heart failure (AHF) is defined as “the new onset or recurrence of symptoms and signs of HF requiring urgent or emergent therapy and resulting in unscheduled care or hospitalization” [1]. It is characterized as the most life-threatening cardiovascular disease causing a huge social and economic burden in both developed and developing countries [2,3]. The prevalence of HF in 2017 was over 5.7 million in the United States and it is estimated that it will rise up to more than 8 million by 2030, accounting for a 46 percent increase in prevalence [4]. The mortality rate within four years in patients with symptomatic HF is 50 percent and only half of those with the end-stage condition survive until the next year, which is much worse than the majority of advanced malignancies [2]. From a clinical standpoint, it is of high importance to distinguish between acute and chronic heart failure; however, it is not always feasible through taking an accurate history, physical exam, and echocardiogram [5]. Therefore, developing objective accurate diagnostic tests has become a main concern in the recent decade. Currently, brain (B-type) natriuretic peptide (BNP) and N-terminal pro-BNP (NT-proBNP) are recommended by the established guidelines for assigning the diagnosis of AHF and determining the prognosis of patients presenting in the emergency department. However, serum levels of BNP and NT-proBNP are affected by a series of conditions such as obesity, age, renal function, atrial fibrillation, thromboembolic events, etc. [6,7]. Also, a multicenter trial investigating a considerable number of patients - approximately 1,100 high-risk HF patients - failed to establish any beneficial effect of using NT-proBNP-guided strategy in the routine outpatient management of these patients [8]. Therefore, investigations are still continued to find other biomarkers with higher accuracy and clinical applicability. Recently, many research studies have demonstrated that micro-ribonucleic acids (miRNAs) can exert diagnostic roles in a variety of conditions including AHF. There is also some evidence advocating the prognostic and therapeutic role of these biologic agents. As Figure-1 demonstrates, the related publication on the association between miRNA and HF has grown considerably since 2007. Therefore, different roles of miRNAs in AHF will be discussed in the following sections.

###  Structure of MicroRNAs

**Figure 2:**
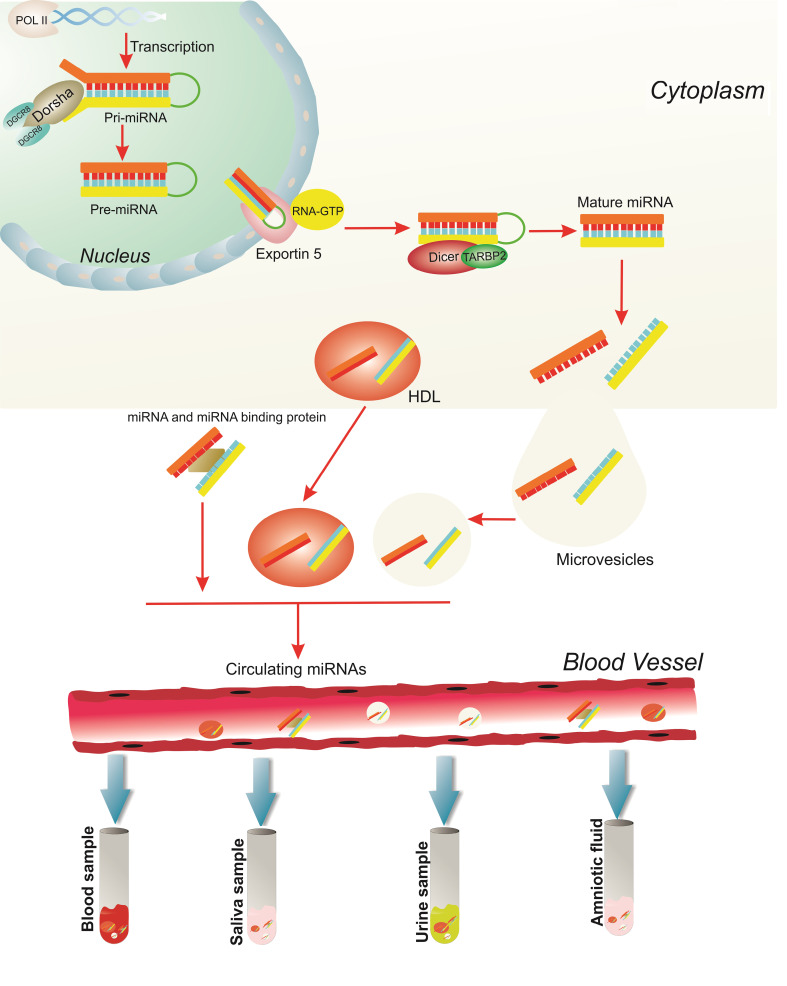
Biogenesis and pathways of circulating miRNAs **. **After transcription from source DNA into pri-miRNAs (with the length of hundreds or thousands of nucleotides), pri-miRNAs are cleaved by Drosha (RNase III enzyme) into preliminary miRNAs with 70- to 100- nucleotide-long (pre-miRNAs) to be able to be transported by exportin-5 to the cytoplasm. In the cytoplasm, the Dicer enzyme cleaves the pre-miRNAs and generates double-stranded immature miRNA duplexes. These 21 to 25-nucleotide length immature miRNA duplexes then become unwounded and by associating with Argonaute proteins generate the RNA-induced silencing complex (RISC). The RISC can regulate the fundamental functions of cells through the degradation of complementary messenger-RNAs (mRNA) of cells’ functional proteins and repression of translation [17,18].

Generally, miRNAs are some small non-coding RNAs with 21-25-nucleotide length [9]. Since their discovery in 1993 in nematode Caenorhabditis elegans and seven years later in humans, their role in different physiological and pathological conditions has been widely studied [10-14]. The role of miRNAs in cardiac development was first described in 2005 by Kwon *et al*. [15]. As completely elucidated by Ali Sheikh *et al*., miRNAs take several modifications from inside the nucleus until they enter the bloodstream [16]. After the mature miRNAs enter the bloodstream as circulating miRNAs, they can be detected in a variety of body fluids (Figure-2) [19,20]. Although the secretion mechanism of miRNAs into extracellular space and bloodstream is not completely understood, there are some theories trying to explain the cellular process such as secretion through membrane-bounded vesicles or by means of protein-miRNA complexes (AGO2, NPM1, and HDL) [16].

###  The Role of MiRNAs in the Pathophysiology of Cardiovascular Disease

Rooij *et al*. in 2006 revealed that miR-195 was significantly increased in the failing human heart. They also demonstrated that miR-195 has an essential role in cardiac remodeling in transgenic mice [21]. Thereafter, a variety of biologic effects of miRNAs in the cardiovascular system have been introduced by further studies. Table-1 summarizes some of the most cited miRNAs having a significant role in the cardiovascular system. Some miRNAs are reported to play roles in cardiac development including miRNAs -1, -27, -130a, -133, and -199a [22-26]; however, their role is not limited to development of heart muscle and they have essential effects on both physiological and pathologic mechanisms in the cardiovascular system. Ten miRNAs are revealed to be mainly involved in cardiac hypertrophy (Table-1). Some of them are related to the pathophysiology of multiple disorders such as miRNA -1 and -133. Both miRNA-1 and -133 have been identified to target key molecules in the signaling pathways relating to cardiac hypertrophy, cardiac fibrosis, and arrhythmia [24,27-29]. In vitro and in vivo studies suggest that the possible mechanism that miRNA-1 may contribute to cardiac hypertrophy is through its role in cell mass and reduced level of miRNA-1 expression is necessary for cell mass increase [23,24]. Overexpression of miRNA-1 can bring about arrhythmia. This is possibly due to its regulatory effect on intracellular calcium level balance and consequently altering the automaticity and cardiac conductance [30]. The miRNA-133 has a protective role against cardiac hypertrophy by some mechanisms such as intracellular calcium concentrations regulation and reduction of ANP and myosin heavy chain beta MHC-β by suppression of their mRNAs’ translation. Changes in MHC-β can lead to cardiac hypertrophy, fibrosis, and contractile function impairment [31,32]. The miRNA-208a is reported to be associated with the increase of MHC-β in cardiomyocytes [33,34]. Silencing miRNA-208 in neonatal mice model of myocardial infraction inhibited apoptosis, hypertrophy, and fibrosis; and it improved cardiac function [35]. The miR-21 plays an important role in the pathophysiology of cardiac fibrosis, hypertrophy, and apoptosis [36-39]. It is preferentially expressed in cardiac fibroblasts and its role in cardiac hypertrophy is reported to be due to stimulation of mitogen-activated protein (MAP) kinases in fibroblasts. The miRNA-195 is claimed to be sufficient for inducing pathological cardiac hypertrophy and heart failure in mice model [21]. There is evidence that miRNA-223 attenuates cardiac hypertrophy by reducing intracellular calcium concentration, inhibiting cardiomyocyte contractility, and phosphorylating cardiac troponin I (cTNI) [40]. Also, other miRNAs such as miRNA-124 and -499 have significant influence in cardiac hypertrophy by inducing angiotensin II-induced hypertrophy and altered expression of contractile proteins, respectively [41,42]. More than twenty different miRNAs are reported to be involved in cardiac fibrosis. These miRNAs exert their pro- or anti-fibrotic effects by means of a variety of mechanisms. The miRNA-133 and -30 modulate the expression of connective tissue growth factor (CTGF), which is a known as an essential mediator in the fibrotic process and the extracellular matrix synthesis [30,43]. The effect of miRNA-133 and -30 by reduction of CTGF significantly reduces collagen deposition [43]. The miRNA-98 inhibits TGF-β1-induced fibrosis in human cardiac fibroblasts [44]. The miRNA-24 prevents fibrosis by inhibiting differentiation and migration of cardiac fibroblasts [45]. In infarcted and failing heart tissue, miRNA-29 is expressed in fibroblasts, resulting in a reduction of collagen expression and inhibiting fibrosis [46]. However, miRNA-203 and -21 have a pro-fibrogenic role. He *et al*., in an in vitro study, demonstrated that miR-203 increases the synthesis of CTGF, transforming growth factor-beta 1 (TGF-β1), and fibronectin [47]. The contribution of miRNAs in different pathologic and physiologic cellular processes relating to HF makes them interesting targets for efficiently regulating gene expression, signaling pathways, cellular functions, and consequently heart muscle function. The changes in the level of miRNAs in body fluids reflect the intracellular events relating to the role of miRNAs in cardiomyocytes or other types of heart-related cells such as cardiac fibroblasts. Accordingly, a diagnostic role of miRNAs is also conceivable which will be investigated in the following section.

###  MicroRNAs and Diagnosis of AHF

Considering some favorable characteristics of miRNAs, they have become a new hope in the diagnosis of heart failure. From a laboratory aspect, appropriate stability of miRNA against severe conditions such as boiling, freezing, extreme pH levels has been well-established suggesting the feasibility of laboratory evaluation in the obtained samples from patients [48,49]. Also, from a clinical aspect, it is postulated that unlike NT-proBNP and BNP, circulating miRNA levels are not affected by clinical confounders including age, sex, body mass index, kidney function, and blood pressure [50]. Besides, they are easily detectable from a variety of body fluids such as blood, urine, saliva, etc. Moreover, due to their special structure made up of a sequence of nucleotides and tissue or pathology specific feature, they can be followed to find the exact source [51]. Therefore, they potentially can serve as excellent noninvasive diagnostic biomarkers in cardiovascular diseases including HF (19, 20). The diagnostic role of over 80 different miRNAs has been evaluated in patients with HF (Table-2). Some of them are evaluated in only one single subtype of HF including HF with reduced ejection fraction (HFrEF) or with preserved ejection fraction (HFpEF). Moreover, HF has been assessed in relationship with different specific miRNAs due to its different etiologies. Additionally, there are pros and cons of association between the level of miRNAs and some of the HF severity-related characteristics including ejection fraction (EF) or laboratory prognostic tests [52]. One of the well-known miRNAs in the diagnosis of HF is miRNA-423-5p which has been suggested by several studies as an excellent biomarker [53-58]. However, Tutarel *et al*. claimed that the level of miRNA-423-5p was not useful to diagnose HFrEF patients [59]. A recent systematic review and meta-analysis of 10 different studies revealed that although miR423-5p had the potential of being a biomarker of HF, BNP was the most convinced biomarker of HF [55]. Several miRNAs are specifically investigated in patients with AHF, the most prominent of which include miRNA- 27, -30, -199, and -423-5p (Table-2) [54,60-63]. Tijsen *et al*. investigated the utility of miRNA-423-5p to discriminate AHF from both healthy controls and non-HF forms of dyspnea. Their results showed miRNA-423-5p can have significant diagnostic value in AHF (area under the curve [AUC] of 0.91). Corsten *et al*. studied the plasma level of a series of miRNAs in patients with AHF comparing them with healthy controls. The results showed all the selected cardiac-related miRNAs including miRNA-208b, -1, -499, and -223 were elevated in AHF patients but only miRNA-499 reached the significance level. Also, a liver-specific miRNA (miRNA-122) was significantly elevated in AHF patients possibly suggesting hepatic venous congestion in this patient group [50]. The diagnostic accuracy of miRNA-499 was reported to be even higher in patients with AHF due to myocardial infarction [64]. Li *et al*. studied plasma levels of circulating miR-302 family members in patients with AHF comparing them to the level of those in AHF-free patients and healthy controls. The results demonstrated that the level of plasma miRNA-302s (except miRNA-302f) was significantly higher in AHF patients with the highest AUC of 0.87 for miRNA-302b-3p. Moreover, they revealed a strong positive correlation between miRNA-302s and NT-proBNP levels [65]. Ovchinnikova *et al*. revealed diagnostic potential of 12 miRNAs for AHF, which only seven of them passed the Bonferroni correction threshold (including miRNA-652-3p, -199a-3p, -106a-5p, -30e-5p, -27a-3p, -26b-5p, and -18a-5p) [66]. The diagnostic capability of these miRNAs was lately confirmed by further analysis yielding the AUC within a range of 0.82–0.97. The highest AUC belonged to miRNA-199a-3p and miRNA-18a-5p [60]. Recently, Wu *et al*. investigated three different miRNAs (including exo-miRNA-92b-5p, -192-5p, and -320a) in patients with AHF due to dilated cardiomyopathy (DCM) as compared to healthy volunteers. The analysis showed that serum exo-miRNA-92b-5p expression was significantly increased in AHF patients. They also demonstrated a diagnostic potential of miRNA-92b-5 for AHF with AUC of 0.808 and sensitivity and specificity of 62.8% and 85.3 %, respectively [67]. However, Seronde *et al*. analyzed the level of selected miRNAs (including miRNA -1, -21, -23, -126, and -423-5p) at the admission of a cohort study and reported that although miRNA-1, -126, and -423-5p were significantly low in AHF compared to non-AHF, none of the selected miRNAs (AUCs<0.70) or their combinations (AUCs<0.75) had considerable diagnostic value for AHF [68]. Apparently, there is little consensus on the capacity of miRNA for being considered as the sole biomarker of AHF. Therefore, some studies suggest the combination of miRNAs and BNP or NT-proBNP as the best strategy of using current biomarkers [55,61]. In a study by Ellis *et al*., a combined assessment of miRNAs and NT-proBNP significantly improved the AUC of NT-proBNP in the diagnosis of AHF by 4.6% [61]. This idea has also been established for non-acute HF [69]. Apparently, miRNAs have more to offer rather than only to diagnose AHF. It seems that the evaluation of miRNAs in patients with possible AHF can be considered not only to diagnose HF but also to evaluate the possible underlying etiology of AHF [70]. Being aware of the underlying cause of AHF can improve the management of these patients. Ikeda *et al*. studied the patterns of miRNAs expression in patients with ischemic cardiomyopathy (ICM), aortic stenosis, and idiopathic cardiomyopathy. The results showed that various types of miRNAs are regulated differentially in each underlying condition [71]. Also, Sucharov *et al*. reported similar results demonstrating that miRNAs had a distinct expression in two types of heart failure including idiopathic dilated cardiomyopathy (DCM) and ICM [72]. Another study revealed that miRNA-499-5p was significantly higher in patients having AHF due to non-ST elevation myocardial infarction than other AHF patients [64]. Some studies advocate the correlation between the severity of AHF and the level of miRNA. Danowski *et al*. reported that the level of serum miRNA-133 significantly decreased with increased severity of AHF in terms of both the New York Heart Association (NYHA) functional class and pulmonary artery occlusion pressure [73]. Wong *et al*. demonstrated that using an 8-microRNA discovery panel can distinguish HF patients with HFrEF and HFpEF [69]. Also, Li *et al*. demonstrated the same capacity for miRNA-302 [65]. Furthermore, Tijsen *et al*. postulated that miRNA-423-5p and miRNA-675 were higher in atherosclerotic forms of AHF as compared to non-atherosclerotic forms of AHF [56].

## Prognosis

Prognosis is essential in the management of HF patients and decision making. Although a variety of miRNAs are discovered to have a diagnostic role in HF, few studies have reported clinically applicable prognostic significance of miRNAs [74,75]. Yet, some miRNAs are demonstrated to have substantial prognostic values such as miRNA-423-5p. In a study by Seronde *et al*., it was observed that miRNA-423-5p could properly predict one-year hospital re-admission (adjusted odds ratio [OR], 0.70) and one-year mortality (OR, 0.54). Patients with lower serum miRNA-423-5p had a significantly higher risk of long-term mortality [68]. Also, Schneider *et al*. evaluated AHF patients by measuring the level of selected miRNAs in several stages including within 24 h of hospital admission, at the time of hospital discharge, and a few weeks post-discharge, and following them for a period of two years. The results demonstrated that patients with higher miRNA4235p level between the time of admission and clinical compensation experienced fewer hospital readmissions in two years. Moreover, in this study, increased levels of miRNA-21 and -126 at the time of clinical compensation predicted better two-year survival and fewer readmissions [76]. Another study revealed that the prognostic value of miRNA-182 was even higher than NT-proBNP and high-sensitivity C-reactive protein (AUC were 0.695, 0.350, and 0.475, respectively). Further analysis by Cox regression yielded a significant predictive value of miRNA-182 for cardiovascular mortality [76]. Xiao *et al*. reported that miRNA-30d can independently predict one-year-all-cause mortality in AHF patients and it can be considered as a prognostic biomarker in AHF (AUC=0.806) [77]. Vegter *et al*. demonstrated that seven miRNAs were significantly negatively correlated to some previously recognized prognostic biomarkers; miRNA-16-5p was correlated to C-reactive protein, miRNA-106a-5p to creatinine, miRNA-223-3p to growth differentiation factor 15, miRNA-652-3p to soluble ST-2, miRNA-199a-3p to procalcitonin, and galectin-3 and miR-18a-5p to procalcitonin [60]. They also previously reported that lower levels of seven miRNAs (including miRNA-let-7i-5p, -18a-5p, -18b-5p, -223-3p, -301a-3p, -423-5p, and -652-3p) two days after AHF admission were in significant association with high risk of 180-day mortality, although only miRNA-18a-5p and -652-3p passed Bonferroni correction in the AHF validation cohort [66]. Other than cardiac-related prognostic characteristics, miRNAs can also predict renal functions in AHF patients. This was revealed by Bruno *et al*. investigating the association between 12 miRNAs and renal function in AHF patients from admission time until three days later. Among several miRNAs (including miRNA-199a-3p, -27a-3p, -652-3p, -423-5p, and -let-7i-5p) which were associated with increase in creatinine during the first 3 days of hospitalization, miRNA-199a-3p was the strongest predictor of renal dysfunction (OR, 1.48 and a C-index, 0.701) [78].

###  Therapeutic Role of MiRNAs 

As described previously in this review, miRNAs play a substantial role in the pathophysiology of HF. Therefore, the therapeutic role of miRNAs in a variety of diseases including HF has become an interesting topic of recent studies and a novel approach in target therapy. Two different techniques are mostly applied for altering the level of miRNA expression within the cells including using antagomirs and miRNA-mimics in order to decrease or increase target miRNAs expression, respectively [79,80]. A study by Hullinger *et al*. revealed that systemic delivery of miRNA-15 antagomir in myocardial infarction models reduces the infarct size and cardiac remodeling and improves cardiac function [81]. Moreover, Montgomery *et al*. reported that systematic delivery of antagomir against miRNA-208a in hypertension-induced HF in Dahl rats could diminish pathological myosin switching, reduce cardiac remodeling, recover cardiac function, and also increase overall survival [82]. On the other hand, Suckau *et al*. used miRNA-mimics to reduce cardiomyocytes’ diameter, cardiac fibrosis, dilation, and hypertrophy. In this in vivo study, RNA interference was delivered by adeno-associated virus vector, which was intravenously injected to target the heart of a rat model of pressure overload. This intervention restored systolic functional parameters to normal ranges [83]. In vitro and in vivo investigations of the effect of miRNA-29b in heart of mice demonstrated that enhancing the activity of miRNA-29b can prevent angiotensin II-mediated cardiac fibrosis and cardiac dysfunction. They also found that this positive effect is through targeting the TGF-β/Smad3 pathway by miRNA-29b [84]. Kumarswamy *et al*. demonstrated that sarcoplasmic reticulum calcium ATPase 2a gene therapy through an Akt/FoxO3A-dependent pathway can normalize the level of mi-RNA-1 in a chronic rat HF model resulting in rescued cardiac function [85]. Another study using antagomir against miRNA-132 improved cardiac hypertrophy and HF in mice, suggesting a possible therapeutic approach [86]. As far as the researchers of this study investigated, no clinical trial has investigated the effect of therapeutic regulation of miRNAs in AHF patients yet. However, there are some in vivo and in vitro studies supporting the idea of such treatment. A series of studies have proposed the beneficial effect of inhibition of miRNA-24 in AHF due to ischemic events [87-90]. Inhibition of miRNA-24 limited myocardial infarct size in acute myocardial infarction and cardiac dysfunction mouse model by inhibiting the endothelial apoptosis and improvement of vascularity, which enhanced cardiac function and survival [87]. Also, adenovirus-directed overexpression of miR-24 in cardiomyocytes reduced both JP2 expression and Ca2+ transient amplitude and improved excitation-contraction coupling in heart cells, suggesting a novel possible strategy in the treatment of HF [91]. Some studies demonstrate different effects of regulation of a single miRNA in HF experimental models. Also, an in vivo study by Thum *et al*. demonstrated that inhibition of miRNA-21 in a pressure-overload-induced disease model mouse could significantly suppress interstitial fibrosis and ameliorate cardiac dysfunction by inhibition of cardiac ERK–MAP kinase activity [36]. On the other hand, Liu *et al*. showed that Trimetazidine leads to reduced right-ventricular cardiomyocytes apoptosis, improved ventricular function, and reduced fibrosis through increasing the expression of miRNA-21 [92]. Furthermore, considering a wide range of miRNA functions in multiple organs, systemic treatment with antagomirs and miRNA-mimics in HF patients may affect other organs, as well. For instance, some of the miRNAs associated with cardiovascular diseases are involved in neoplasms as tumor suppressors [93]. Therefore, targeting miRNAs necessitate a thorough evaluation of parallel mechanisms in other cells and tissues to prevent adverse effects.

## Conclusion

 Generally, it has been demonstrated that miRNAs play an essential role in the pathophysiology of HF. Furthermore, many studies have postulated the great potential of miRNAs to be used as diagnostic biomarkers in AHF. Although there is not a proper consensus regarding which miRNA is the best for such implication, the diagnostic applicability of some miRNAs such as miRNA-423-5p have been verified by many studies but not completely successful in comparison with NT-proBNP as the most acknowledged HF biomarker. The prognostic efficacy of miRNA-423-5p and some other miRNAs are also suggested by a variety of studies; however, an adequate consensus is also lacking for this application. Although invaluable attempts have been accomplished regarding therapeutic regulation of miRNAs and these mediators have become future hopes for the treatment of AHF, extensive investigations are still necessary to advance them up to the clinical application.

## Acknowledgement

 This study was supported by a fund from the Tabriz University of Medical Sciences.

## Conflict of Interest

 The authors declare that they have no conflict of interests.

**Table 1:** Discovered Functions of Some Prominent MiRNAs in the Cardiovascular System

**Role in the Cardiovascular System**	**Related miRNA**	Reference
Cardiac development	1, 27, 130a, 133, 199a	[22-26,37,94-100]
Vascular inflammation	10a,b, 221, 222	[71,101,102]
Apoptosis induction Apoptosis inhibition	15, 16, 21 451	[21,28,103] [104]
Endothelial function modulation	21, 125a,b, 150, 126	[71,103,105-107]
Cardiac hypertrophy	1, 21, 23 a,b, 27a,b, 124, 133, 195, 208, 223, 499	[12,21,24,34,98,108,109]
Angiogenesis inhibition Angiogenesis promotion	17, 21, 34a,b, 92, 132, 221, 222, 214, 195, 499-5p, 27, 101a,b, 126	[21,72,102,110-117] [105,107,117-119]
Regulation of beta-adrenergic receptors	100, 133	[72]
Ischemic injury	1, 22, 320, 374	[25,120-123]
Differentiation of vascular smooth muscle	143, 145	[124]
Cardiac fibrosis	1, 15, 21, 22, 24, 26, 29, 30, 34, 101, 122, 132, 133, 155, 199, 203, 208, 214, 433, 489, 503	[28,36,45,84,125,126-128]
Arrhythmia	1, 26, 30, 130, 133, 208	[27,29,34,129,130]

**Table 2:** The MiRNAs in Diagnosis, Prognosis, and Treatment of Patients with Acute and Chronic Heart Failure Measured in Whole Blood, Plasma, or Serum Samples

			**Diagnosis**			**Severity**	**Prognosis**	**Therapeutic Target**	**Underlying Condition**	**Reference**
**MiRNA**	**AHF**	**CHF **			**HFrEF vs HFpEF**					
			**HFrEF**	**HFpEF**						
1	-/+					-	-		Hypertensive or DCM	[50,61,68,131,132]
7									DCM	[131]
let-7i-5p	+						+++			[60,78,133]
9									Hypertensive	[134]
16	+						+		Hypertensive	[54,60]
17									ICM	[135,136]
18a-5p	++						++			[60,66]
19									Congenital	[62]
20									Hypertensive or ICM	[54,134]
21		++				+	-/+		Hypertensive or ICM	[61,68,76,135,132,137,138]
22			+				+			[53]
23a	+				-		-			[61]
24								+++	Diabetic	[89,90,139]
26	++		+				++		Hypertensive	[60,66,134,140]
27	+++				-		++		congenital	[60-62,66,78,119]
29			++						DCM or congenital	[131,140,141]
30	+++	++	+		+		+++			[60,63,66,77,140,142]
34		+							Diabetic or CAD	[143,144]
65							+			[78]
92			++				+		ICM	[53,136,140]
93									Hypertensive	[54]
99									DCM	[145]
103	+				-				Congenital	[61,146]
106	++						++		Hypertensive or ICM	[54,60,66,135]
122	+	+	+			+				[50,52,137]
125a			+		+				ICM or DCM	[135,147]
126	+	+				++	-/+		Hypertensive or ICM	[68,76,134,136,148-150]
130									Congenital	[62]
133	-					+			Hypertensive or Diabetic	[17,73,134,107,151]
142-3p	+			+	-					[61,152]
143									Hypertensive	[134]
145			+						Hypertensive or DCM	[131,134,140]
146		+			+					[63]
147									DCM	[145]
155		+			-				ICM, DCM, or Congenital	[136,137,145,146,153]
181									DCM	[131]
182		+					+		CAD	[137,154]
183-3p		+	+	+						[147]
190a		+		+	+					[147]
192		+								[144]
193		+	+							[147]
194		+							DCM	[144,145]
198									Congenital	[62]
199	+++				-		+++		Diabetic	[60,61,66,143]
205									DCM	[78,145]
208	-	+						+	Hypertensive or CAD	[50,82,132,155]
210		+							Diabetic	[142,143]
211-5p		+	+							[147]
214									DCM	[131]
218									DCM	[145]
221		+			+					[63]
223		+					+		Hypertensive or Diabetic	[54,60,143]
301							+			[78]
302	+				+	+			ICM and DCM	[65,135,145]
320a			+				+			[53]
324-5p	+				-					[61]
328		+			+					[63]
342	+				-				DCM	[61,131]
361		+							ICM	[156]
375		+			+					[63]
378									DCM	[131]
423-5p	+++	+	++			+	+++		Hypertensive or Atherosclerotic or DCM	[53-58,60,68,76,78]
454				+					DCM	[145,152]
486									Congenital	[146]
494		+	+							[147]
499	++	+							Hypertensive or CAD	[50,64,132,155]
500				+						[152]
518		+							DCM	[137,145]
520d-5p			+			+				[52]
544									DCM	[145]
548			+							[64]
550-5p				+	+					[147]
558			+			+				[52]
618									DCM	[145]
636									DCM	[153]
638			+		+					[147]
639									DCM	[153]
646									DCM	[153]
652	++						+++			[60,66,78]
671		+	+							[147]
875									DCM	[145]
1187		+							ICM	[156]
1233		+		+						[147]
1246				+						[152]
1260									Congenital	[146]
1306							+			[157]

Reported to have significant role in + : one study, ++ : two studies, +++ : three or more studies. – : reported having no significant role. +/- : some studies confirm and some reject its role. miRNA: micro RNA, AHF: Acute heart failure, CHF: Chronic heart failure, HFrEF: Heart failure with reduced ejection fraction, HFpEF: Heart failure with preserved ejection fraction, DCM: Dilated cardiomyopathy, ICM: ischemic cardiomyopathy, CAD: Coronary artery disease

## References

[1] Zipes DP, Libby P, Bonow RO, Mann DL, Tomaselli GF. Braunwald's Heart Disease A Textbook of Cardiovascular Medicine. Elsevier Health Sciences; 2018.

[2] Yancy CW, Jessup M, Bozkurt B (2013). for the Task Force on Practice Guidelines 2013 ACCF/AHA guideline for the management of heart failure: executive summary: a report of the American College of Cardiology Foundation/American Heart Association. J Am Coll Cardiol.

[3] von Lueder TG, Agewall S (2018). The burden of heart failure in the general population: a clearer and more concerning picture. J Thorac Dis.

[4] Savarese G, Lund LH (2017). Global Public Health Burden of Heart Failure. Heart Fail Rev.

[5] Daniels SR (2006). Acute vs chronic heart failure. J Pediatr.

[6] Richards M, Di Somma S, Mueller C, Nowak R, Peacock WF (2013). Atrial fibrillation impairs the diagnostic performance of cardiac natriuretic peptides in dyspneic patients: results from the BACH Study (Biomarkers in ACute Heart Failure). JACC: Heart Failure.

[7] Christenson RH, Azzazy HME, Duh S-H, Maynard S, Seliger SL, Defilippi CR (2010). Impact of increased body mass index on accuracy of B-type natriuretic peptide (BNP) and N-terminal proBNP for diagnosis of decompensated heart failure and prediction of all-cause mortality. Clin Chem.

[8] Felker GM, Ahmad T, Anstrom KJ, Adams KF, Cooper LS (2014). Rationale and design of the GUIDE-IT study: guiding evidence based therapy using biomarker intensified treatment in heart failure. JACC: Heart Failure.

[9] Tao J, Li SF, Xu M (2011). [The roles of microRNA in the diagnosis and therapy for cardiovascular diseases]. [Progress in physiology].

[10] Reddy KB (2015). MicroRNA (miRNA) in cancer. Cancer Cell Int.

[11] Ludwig N, Leidinger P, Becker K, Backes C, Fehlmann T (2016). Distribution of miRNA expression across human tissues. Nucleic Acids Res.

[12] Chakraborty C, Sharma AR, Sharma G, Doss CGP, Lee S-S (2017). Therapeutic miRNA and siRNA: moving from bench to clinic as next generation medicine. Molecular Therapy-Nucleic Acids.

[13] Jeon T-I, Osborne TF (2016). miRNA and cholesterol homeostasis. Biochimica et Biophysica Acta (BBA)-Molecular and Cell Biology of Lipids.

[14] Lee RC, Feinbaum RL, Ambros V (1993). The C elegans heterochronic gene lin-4 encodes small RNAs with antisense complementarity to lin-14. Cell.

[15] Kwon C, Han Z, Olson EN, Srivastava D (2005). MicroRNA1 influences cardiac differentiation in Drosophila and regulates Notch signaling. Proc Natl Acad Sci U S A.

[16] Ali Sheikh MS, Salma U, Zhang B, Chen J, Zhuang J, Ping Z (2016). Diagnostic, Prognostic, and Therapeutic Value of Circulating miRNAs in Heart Failure Patients Associated with Oxidative Stress. Oxid Med Cell Longev.

[17] Romaine SPR, Tomaszewski M, Condorelli G, Samani NJ (2015). MicroRNAs in cardiovascular disease: an introduction for clinicians. Heart.

[18] Condorelli G, Latronico MVG, Cavarretta E (2014). microRNAs in cardiovascular diseases: current knowledge and the road ahead. J Am Coll Cardiol.

[19] Weber JA, Baxter DH, Zhang S, Huang DY, Huang KH (2010). The microRNA spectrum in 12 body fluids. Clin Chem.

[20] Cortez MA, Bueso-Ramos C, Ferdin J, Lopez-Berestein G, Sood AK, Calin GA (2011). MicroRNAs in body fluids—the mix of hormones and biomarkers. Nat Rev Clin Oncol.

[21] Van Rooij E, Sutherland LB, Liu N, Williams AH, McAnally J (2006). A signature pattern of stress-responsive microRNAs that can evoke cardiac hypertrophy and heart failure. Proc Natl Acad Sci U S A.

[22] Sayed D, Hong C, Chen I-Y, Lypowy J, Abdellatif M (2007). MicroRNAs play an essential role in the development of cardiac hypertrophy. Circ Res.

[23] Elia L, Contu R, Quintavalle M, Varrone F, Chimenti C (2009). Reciprocal regulation of microRNA-1 and insulin-like growth factor-1 signal transduction cascade in cardiac and skeletal muscle in physiological and pathological conditions. Circulation.

[24] Care A, Catalucci D, Felicetti F, Bonci D, Addario A (2007). MicroRNA-133 controls cardiac hypertrophy. Nat Med.

[25] Pan Z, Sun X, Ren J, Li X, Gao X (2012). miR-1 exacerbates cardiac ischemia-reperfusion injury in mouse models. PLoS One.

[26] Lozano-Velasco E, Galiano-Torres J, Jodar-Garcia A, Aranega AE, Franco D (2015). miR-27 and miR-125 distinctly regulate muscle-enriched transcription factors in cardiac and skeletal myocytes. BioMed research international.

[27] Li H, Li S, Yu B, Liu S (2012). Expression of miR-133 and miR-30 in chronic atrial fibrillation in canines. Mol Med Report.

[28] Thum T (2014). Noncoding RNAs and myocardial fibrosis. Nature Reviews Cardiology.

[29] Zhang Y, Sun L, Zhang Y, Liang H, Li X (2013). Overexpression of microRNA-1 causes atrioventricular block in rodents. Int J Biol Sci.

[30] Silva DCPd, Carneiro FD, Almeida KCd, Fernandes-Santos C (2018). Role of miRNAs on the Pathophysiology of Cardiovascular Diseases. Arq Bras Cardiol.

[31] Lee S-Y, Lee CY, Ham O, Moon JY, Lee J (2018). microRNA-133a attenuates cardiomyocyte hypertrophy by targeting PKCδ and Gq. Mol Cell Biochem.

[32] Wu Y, Wang YQ, Wang BX (2013). MicroRNA-133a attenuates isoproterenol-induced neonatal rat cardiomyocyte hypertrophy by downregulating L-type calcium channel α1C subunit gene expression. Arq Bras Cardiol.

[33] Stelzer JE, Brickson SL, Locher MR, Moss RL (2007). Role of myosin heavy chain composition in the stretch activation response of rat myocardium. J Physiol Pharmacol.

[34] Callis TE, Pandya K, Seok HY, Tang R-H, Tatsuguchi M (2009). MicroRNA-208a is a regulator of cardiac hypertrophy and conduction in mice. J Clin Invest.

[35] Tony H, Meng K, Wu B, Yu A, Zeng Q (2015). MicroRNA-208a dysregulates apoptosis genes expression and promotes cardiomyocyte apoptosis during ischemia and its silencing improves cardiac function after myocardial infarction. Mediators Inflamm.

[36] Thum T, Gross C, Fiedler J, Fischer T, Kissler S (2008). MicroRNA-21 contributes to myocardial disease by stimulating MAP kinase signalling in fibroblasts. Nature.

[37] Da Costa Martins PA, De Windt LJ (2012). MicroRNAs in control of cardiac hypertrophy. Cardiovasc Res.

[38] Sayed D, He M, Hong C, Gao S, Rane S (2010). MicroRNA-21 is a downstream effector of AKT that mediates its antiapoptotic effects via suppression of Fas ligand. J Biol Chem.

[39] Wong LL, Wang J, Liew OW, Richards AM, Chen YT (2016). MicroRNA and Heart Failure. Int J Mol Sci.

[40] Wang Y-S, Zhou J, Hong K, Cheng X-S, Li Y-G (2015). MicroRNA-223 displays a protective role against cardiomyocyte hypertrophy by targeting cardiac troponin I-interacting kinase. Cell Physiol Biochem.

[41] Bao Q, Chen L, Li J, Zhao M, Wu S (2017). Role of microRNA-124 in cardiomyocyte hypertrophy inducedby angiotensin II. Cell Mol Biol.

[42] Shieh JTC, Huang Y, Gilmore J, Srivastava D (2011). Elevated miR-499 levels blunt the cardiac stress response. PLoS One.

[43] Duisters RF, Tijsen AJ, Schroen B, Leenders JJ, Lentink V (2009). miR-133 and miR-30 regulate connective tissue growth factor: implications for a role of microRNAs in myocardial matrix remodeling. Circ Res.

[44] Cheng R, Dang R, Zhou Y, Ding M, Hua H (2017). MicroRNA-98 inhibits TGF-β1-induced differentiation and collagen production of cardiac fibroblasts by targeting TGFBR1. Hum Cell.

[45] Wang J, Huang W, Xu R, Nie Y, Cao X (2012). Micro RNA‐24 regulates cardiac fibrosis after myocardial infarction. J Cell Mol Med.

[46] Lai K-B, Sanderson JE, Izzat MB, Yu C-M (2015). Micro-RNA and mRNA myocardial tissue expression in biopsy specimen from patients with heart failure. Int J Cardiol.

[47] He Q, Wang CM, Qin JY, Zhang YJ, Xia DS (2017). Effect of miR-203 expression on myocardial fibrosis. Eur Rev Med Pharmacol Sci.

[48] Zhu W, Qin W, Atasoy U, Sauter ER (2009). Circulating microRNAs in breast cancer and healthy subjects. BMC Res Notes.

[49] Patnaik SK, Mallick R, Yendamuri S (2010). Detection of microRNAs in dried serum blots. Anal Biochem.

[50] Corsten MF, Dennert R, Jochems S, Kuznetsova T, Devaux Y (2010). Circulating MicroRNA-208b and MicroRNA-499 reflect myocardial damage in cardiovascular disease. Circ Cardiovasc Genet.

[51] Schmitter D, Cotter G, Voors AA (2014). Clinical use of novel biomarkers in heart failure: towards personalized medicine. Heart Fail Rev.

[52] Vogel B, Keller A, Frese KS, Leidinger P, Sedaghat-Hamedani F (2013). Multivariate miRNA signatures as biomarkers for non-ischaemic systolic heart failure. Eur Heart J.

[53] Goren Y, Kushnir M, Zafrir B, Tabak S, Lewis BS, Amir O (2012). Serum levels of microRNAs in patients with heart failure.

[54] Dickinson BA, Semus HM, Montgomery RL, Stack C, Latimer PA (2013). Plasma microRNAs serve as biomarkers of therapeutic efficacy and disease progression in hypertension‐induced heart failure. Eur J Heart Fail.

[55] Yan H, Ma F, Zhang Y, Wang C, Qiu D (2017). miRNAs as biomarkers for diagnosis of heart failure: A systematic review and meta-analysis. Medicine.

[56] Tijsen AJ, Creemers EE, Moerland PD, de Windt LJ, van der Wal AC (2010). MiR423-5p as a circulating biomarker for heart failure. Circ Res.

[57] Fan K-L, Zhang H-F, Shen J, Zhang Q, Li X-L (2013). Circulating microRNAs levels in Chinese heart failure patients caused by dilated cardiomyopathy. Indian Heart J.

[58] Goretti E, Seronde MF, Vausort M, Gayat E, Thum T (2014). Circulating microRNAs and outcome in patients with acute heart failure. Cardiovasc Res.

[59] Tutarel O, Dangwal S, Bretthauer J, Westhoff-Bleck M, Roentgen P (2013). Circulating miR-423_5p fails as a biomarker for systemic ventricular function in adults after atrial repair for transposition of the great arteries. Int J Cardiol.

[60] Vegter EL, Schmitter D, Hagemeijer Y, Ovchinnikova ES, van der Harst P (2016). Use of biomarkers to establish potential role and function of circulating microRNAs in acute heart failure. Int J Cardiol.

[61] Ellis KL, Cameron VA, Troughton RW, Frampton CM, Ellmers LJ, Richards AM (2013). Circulating microRNAs as candidate markers to distinguish heart failure in breathless patients. Eur J Heart Fail.

[62] Chen W, Li S (2017). Circulating microRNA as a novel biomarker for pulmonary arterial hypertension due to congenital heart disease. Pediatr Cardiol.

[63] Watson CJ, Gupta SK, O'Connell E, Thum S, Glezeva N (2015). MicroRNA signatures differentiate preserved from reduced ejection fraction heart failure. Eur J Heart Fail.

[64] Olivieri F, Antonicelli R, Lorenzi M, D'Alessandra Y, Lazzarini R (2013). Diagnostic potential of circulating miR-499-5p in elderly patients with acute non ST-elevation myocardial infarction. Int J Cardiol.

[65] Li G, Song Y, Li YD, Jie LJ, Wu WY (2018). Circulating miRNA-302 family members as potential biomarkers for the diagnosis of acute heart failure. Biomark Med.

[66] Ovchinnikova ES, Schmitter D, Vegter EL, ter Maaten JM, Valente MAE (2016). Signature of circulating microRNAs in patients with acute heart failure. Eur J Heart Fail.

[67] Wu T, Chen Y, Du Y, Tao J, Zhou Z, Yang Z (2018). Serum Exosomal MiR-92b-5p as a Potential Biomarker for Acute Heart Failure Caused by Dilated Cardiomyopathy. Cell Physiol Biochem.

[68] Seronde M-F, Vausort M, Gayat E, Goretti E, Ng LL (2015). Circulating microRNAs and outcome in patients with acute heart failure. PLoS One.

[69] Wong LL, Zou R, Zhou L, Lim JY, Phua DCY (2019). Combining Circulating MicroRNA and NT-proBNP to Detect and Categorize Heart Failure Subtypes. J Am Coll Cardiol.

[70] Oliveira-Carvalho V, Da Silva MMF, Guimaraes GV, Bacal F, Bocchi EA (2013). MicroRNAs: new players in heart failure. Molecular biology reports.

[71] Ikeda S, Kong SW, Lu J, Bisping E, Zhang H (2007). Altered microRNA expression in human heart disease. Physiol Genomics.

[72] Sucharov C, Bristow MR, Port JD (2008). miRNA expression in the failing human heart: Functional correlates. J Mol Cell Cardiol.

[73] Danowski N, Manthey I, Jakob HG, Siffert W, Peters J, Frey UH (2013). Decreased Expression of miR-133a but Not of miR-1 is Associated with Signs of Heart Failure in Patients Undergoing Coronary Bypass Surgery. Cardiology.

[74] Weckbach LT, Grabmaier U, Clauss S, Wakili R (2016). MicroRNAs as a diagnostic tool for heart failure and atrial fibrillation. Curr Opin Pharmacol.

[75] Pourafkari L, Tajlil A, Nader ND (2019). Biomarkers in diagnosing and treatment of acute heart failure. Biomark Med.

[76] Schneider S, Silvello D, Martinelli NC, Garbin A, Biolo A (2018). Plasma levels of microRNA-21, -126 and -423-5p alter during clinical improvement and are associated with the prognosis of acute heart failure. Mol Med Rep.

[77] Xiao J, Gao R, Bei Y, Zhou Q, Zhou Y (2017). Circulating miR-30d predicts survival in patients with acute heart failure. Cell Physiol Biochem.

[78] Bruno N, ter Maaten JM, Ovchinnikova ES, Vegter EL, Valente MAE (2016). MicroRNAs relate to early worsening of renal function in patients with acute heart failure. Int J Cardiol.

[79] Wang Z. The guideline of the design and validation of MiRNA mimics. MicroRNA and Cancer. Springer; 2011. p. 211-23. 10.1007/978-1-60761-863-8_15 20931400

[80] Krützfeldt J, Rajewsky N, Braich R, Rajeev KG, Tuschl T (2005). Silencing of microRNAs in vivo with ‘antagomirs’. Nature.

[81] Hullinger TG, Montgomery RL, Seto AG, Dickinson BA, Semus HM (2012). Inhibition of miR-15 protects against cardiac ischemic injury. Circ Res.

[82] Montgomery RL, Hullinger TG, Semus HM, Dickinson BA, Seto AG (2011). Therapeutic inhibition of miR-208a improves cardiac function and survival during heart failure. Circulation.

[83] Suckau L, Fechner H, Chemaly E, Krohn S, Hadri L (2009). Long-term cardiac-targeted RNA interference for the treatment of heart failure restores cardiac function and reduces pathological hypertrophy. Circulation.

[84] Zhang Y, Huang X-R, Wei L-H, Chung ACK, Yu C-M, Lan H-Y (2014). miR-29b as a Therapeutic Agent for Angiotensin II-induced Cardiac Fibrosis by Targeting TGF-β/Smad3 signaling. Mol Ther.

[85] Kumarswamy R, Lyon AR, Volkmann I, Mills AM, Bretthauer J (2012). SERCA2a gene therapy restores microRNA-1 expression in heart failure via an Akt/FoxO3A-dependent pathway. Eur Heart J.

[86] Ucar A, Gupta SK, Fiedler J, Erikci E, Kardasinski M (2012). The miRNA-212/132 family regulates both cardiac hypertrophy and cardiomyocyte autophagy. Nat Commun.

[87] Fiedler J, Jazbutyte V, Kirchmaier BC, Gupta SK, Lorenzen J (2011). MicroRNA-24 regulates vascularity after myocardial infarction. Circulation.

[88] Pan LJ, Wang X, Ling Y, Gong H (2017). MiR-24 alleviates cardiomyocyte apoptosis after myocardial infarction via targeting BIM. Eur Rev Med Pharmacol Sci.

[89] Xiao X, Lu Z, Lin V, May A, Shaw DH (2018). MicroRNA miR-24-3p Reduces Apoptosis and Regulates Keap1-Nrf2 Pathway in Mouse Cardiomyocytes Responding to Ischemia/Reperfusion Injury. Oxid Med Cell Longev.

[90] Meloni M, Marchetti M, Garner K, Littlejohns B, Sala-Newby G (2013). Local inhibition of microRNA-24 improves reparative angiogenesis and left ventricle remodeling and function in mice with myocardial infarction. Mol Ther.

[91] Xu M, Wu HD, Li RC, Zhang HB, Wang M (2012). Mir-24 regulates junctophilin-2 expression in cardiomyocytes. Circ Res.

[92] Liu F, Yin L, Zhang L, Liu W, Liu J (2012). Trimetazidine improves right ventricular function by increasing miR-21 expression. Int J Mol Med.

[93] Chini VP (2015). Micro-RNAs and next generation sequencing: new perspectives in heart failure. Clin Chim Acta.

[94] Biyashev D, Veliceasa D, Topczewski J, Topczewska JM, Mizgirev I (2012). miR-27b controls venous specification and tip cell fate. Blood.

[95] Pan W, Zhong Y, Cheng C, Liu B, Wang L (2013). MiR-30-regulated autophagy mediates angiotensin II-induced myocardial hypertrophy. PLoS One.

[96] Kim GH, Samant SA, Earley JU, Svensson EC (2009). Translational control of FOG-2 expression in cardiomyocytes by microRNA-130a. PLoS One.

[97] Thum T, Galuppo P, Wolf C, Fiedler J, Kneitz S (2007). MicroRNAs in the human heart. Circulation.

[98] Suckau L, Fechner H, Chemaly E, Krohn S, Hadri L (2009). Long-term cardiac-targeted RNA interference for the treatment of heart failure restores cardiac function and reduces pathological hypertrophy. Circulation.

[99] Duan Q, Chen C, Yang L, Li N, Gong W (2015). MicroRNA regulation of unfolded protein response transcription factor XBP1 in the progression of cardiac hypertrophy and heart failure in vivo. J Transl Med.

[100] Chinchilla A, Lozano E, Daimi H, Esteban FJ, Crist C (2010). MicroRNA profiling during mouse ventricular maturation: a role for miR-27 modulating Mef2c expression. Cardiovasc Res.

[101] Fang Y, Shi C, Manduchi E, Civelek M, Davies PF (2010). MicroRNA-10a regulation of proinflammatory phenotype in athero-susceptible endothelium in vivo and in vitro. Proc Natl Acad Sci U S A.

[102] Zhu N, Zhang D, Chen S, Liu X, Lin L (2011). Endothelial enriched microRNAs regulate angiotensin II-induced endothelial inflammation and migration. Atherosclerosis.

[103] Weber M, Baker MB, Moore JP, Searles CD (2010). MiR-21 is induced in endothelial cells by shear stress and modulates apoptosis and eNOS activity. Biochem Biophys Res Commun.

[104] Zhang X, Wang X, Zhu H, Zhu C, Wang Y (2010). Synergistic effects of the GATA-4-mediated miR-144/451 cluster in protection against simulated ischemia/reperfusion-induced cardiomyocyte death. J Mol Cell Cardiol.

[105] Wang S, Aurora AB, Johnson BA, Qi X, McAnally J (2008). The endothelial-specific microRNA miR-126 governs vascular integrity and angiogenesis. Dev Cell.

[106] Li D, Yang P, Xiong Q, Song X, Yang X (2010). MicroRNA-125a/b-5p inhibits endothelin-1 expression in vascular endothelial cells. J Hypertens.

[107] Fish JE, Santoro MM, Morton SU, Yu S, Yeh R-F (2008). miR-126 regulates angiogenic signaling and vascular integrity. Dev Cell.

[108] Matkovich SJ, Van Booven DJ, Youker KA, Torre-Amione G, Diwan A (2009). Reciprocal regulation of myocardial miR and mRNA in human cardiomyopathy and reversal of the miR signature by biomechanical support. Circulation.

[109] Han M, Toli J, Abdellatif M (2011). MicroRNAs in the cardiovascular system. Current opinion in cardiology.

[110] Zhu H, Yang Y, Wang Y, Li J, Schiller PW, Peng T (2011). MicroRNA-195 promotes palmitate-induced apoptosis in cardiomyocytes by down-regulating Sirt1. Cardiovasc Res.

[111] Zhao T, Li J, Chen AF (2010). MicroRNA-34a induces endothelial progenitor cell senescence and impedes its angiogenesis via suppressing silent information regulator 1. American Journal of Physiology-Endocrinology and Metabolism.

[112] Katare R, Riu F, Mitchell K, Gubernator M, Campagnolo P (2011). Transplantation of human pericyte progenitor cells improves the repair of infarcted heart through activation of an angiogenic program involving micro-RNA-132. Circ Res.

[113] Wang J, Jia Z, Zhang C, Sun M, Wang W (2014). miR-499 protects cardiomyocytes from H2O2-induced apoptosis via its effects on Pdcd4 and Pacs2. RNA Biol.

[114] Zhang B, Zhou M, Li C, Zhou J, Li H (2014). MicroRNA-92a inhibition attenuates hypoxia/reoxygenation-induced myocardiocyte apoptosis by targeting Smad7. PLoS One.

[115] Doebele C, Bonauer A, Fischer A, Scholz A, Reiss Y (2010). Members of the microRNA-17-92 cluster exhibit a cell-intrinsic antiangiogenic function in endothelial cells. Blood.

[116] Bonauer A, Carmona G, Iwasaki M, Mione M, Koyanagi M (2009). MicroRNA-92a controls angiogenesis and functional recovery of ischemic tissues in mice. Science.

[117] Santulli G (2016). MicroRNAs and endothelial (Dys) function. J Cell Physiol.

[118] Smits M, Mir SE, Nilsson RJA, van der Stoop PM, Niers JM (2011). Down-regulation of miR-101 in endothelial cells promotes blood vessel formation through reduced repression of EZH2. PLoS One.

[119] Yuan Y, Zhang Z, Wang Z, Liu J (2019). MiRNA-27b Regulates Angiogenesis by Targeting AMPK in Mouse Ischemic Stroke Model. Neuroscience.

[120] Ren X-P, Wu J, Wang X, Sartor MA, Jones K (2009). MicroRNA-320 is involved in the regulation of cardiac ischemia/reperfusion injury by targeting heat-shock protein 20. Circulation.

[121] Huang ZQ, Xu W, Wu JL, Lu X, Chen XM (2019). MicroRNA-374a protects against myocardial ischemia-reperfusion injury in mice by targeting the MAPK6 pathway. Life Sci.

[122] Chen Z, Qi Y, Gao C (2015). Cardiac myocyte-protective effect of microRNA-22 during ischemia and reperfusion through disrupting the caveolin-3/eNOS signaling. Int J Clin Exp Pathol.

[123] Saif J, Emanueli C (2014). miRNAs in post-ischaemic angiogenesis and vascular remodelling. Biochem Soc Trans.

[124] Cordes KR, Sheehy NT, White MP, Berry EC, Morton SU (2009). miR-145 and miR-143 regulate smooth muscle cell fate and plasticity. Nature.

[125] Zhou Y, Deng L, Zhao D, Chen L, Yao Z (2016). Micro RNA-503 promotes angiotensin II-induced cardiac fibrosis by targeting Apelin-13. J Cell Mol Med.

[126] Tao L, Bei Y, Chen P, Lei Z, Fu S (2016). Crucial role of miR-433 in regulating cardiac fibrosis. Theranostics.

[127] Yuan J, Chen H, Ge D, Xu Y, Xu H (2017). Mir-21 promotes cardiac fibrosis after myocardial infarction via targeting Smad7. Cell Physiol Biochem.

[128] Creemers EE, van Rooij E (2016). Function and therapeutic potential of noncoding RNAs in cardiac fibrosis. Circ Res.

[129] Shan H, Zhang Y, Cai B, Chen X, Fan Y (2013). Upregulation of microRNA-1 and microRNA-133 contributes to arsenic-induced cardiac electrical remodeling. Int J Cardiol.

[130] Osbourne A, Calway T, Broman M, McSharry S, Earley J, Kim GH (2014). Downregulation of connexin43 by microRNA-130a in cardiomyocytes results in cardiac arrhythmias. J Mol Cell Cardiol.

[131] Prasad SVN, Gupta MK, Duan Z-H, Surampudi VSK, Liu C-G (2017). A unique microRNA profile in end-stage heart failure indicates alterations in specific cardiovascular signaling networks. PLoS One.

[132] Kontaraki JE, Marketou ME, Zacharis EA, Parthenakis FI, Vardas PE (2013). MiR-1, miR-9 and miR-126 levels in peripheral blood mononuclear cells of patients with essential hypertension associate with prognostic indices of ambulatory blood pressure monitoring. Eur Heart J.

[133] Vegter EL, Ovchinnikova ES, van Veldhuisen DJ, Jaarsma T, Berezikov E (2017). Low circulating microRNA levels in heart failure patients are associated with atherosclerotic disease and cardiovascular-related rehospitalizations. Clin Res Cardiol.

[134] Kontaraki JE, Marketou ME, Zacharis EA, Parthenakis FI, Vardas PE (2013). Mir-143/mir-145 levels in peripheral blood mononuclear cells associate with ambulatory blood pressure monitoring parameters in patients with essential hypertension. Eur Heart J.

[135] Li X, Liu CY, Li YS, Xu J, Li DG (2016). Deep RNA sequencing elucidates microRNA-regulated molecular pathways in ischemic cardiomyopathy and nonischemic cardiomyopathy. Genet Mol Res.

[136] Fichtlscherer S, De Rosa S, Fox H, Schwietz T, Fischer A (2010). Circulating microRNAs in patients with coronary artery disease. Circ Res.

[137] Cakmak HA, Coskunpinar E, Ikitimur B, Barman HA, Karadag B (2015). The prognostic value of circulating microRNAs in heart failure: preliminary results from a genome-wide expression study. J Cardiovasc Med.

[138] Zhang J, Xing Q, Zhou X, Li J, Li Y (2017). Circulating miRNA-21 is a promising biomarker for heart failure. Mol Med Report.

[139] Deng X, Liu Y, Luo M, Wu J, Ma R (2017). Circulating miRNA-24 and its target YKL-40 as potential biomarkers in patients with coronary heart disease and type 2 diabetes mellitus. Oncotarget.

[140] Marfella R, Di Filippo C, Potenza N, Sardu C, Rizzo MR (2013). Circulating microRNA changes in heart failure patients treated with cardiac resynchronization therapy: responders vs non-responders. Eur J Heart Fail.

[141] Dawson K, Wakili R, Ördög B, Clauss S, Chen Y (2013). MicroRNA29: a mechanistic contributor and potential biomarker in atrial fibrillation. Circulation.

[142] Zhao DS, Chen Y, Jiang H, Lu JP, Zhang G (2013). Serum miR-210 and miR-30a expressions tend to revert to fetal levels in Chinese adult patients with chronic heart failure. Cardiovasc Pathol.

[143] Greco S, Fasanaro P, Castelvecchio S, D’Alessandra Y, Arcelli D (2012). MicroRNA dysregulation in diabetic ischemic heart failure patients. Diabetes.

[144] Matsumoto S, Sakata Y, Suna S, Nakatani D, Usami M (2013). Circulating p53-responsive microRNAs are predictive indicators of heart failure after acute myocardial infarction. Circ Res.

[145] Enes Coşkun M, Kervancıoğlu M, Öztuzcu S, Yılmaz Coşkun F, Ergün S (2016). Plasma microRNA profiling of children with idiopathic dilated cardiomyopathy. Biomarkers.

[146] Mukai N, Nakayama Y, Murakami S, Tanahashi T, Sessler DI (2018). Potential contribution of erythrocyte microRNA to secondary erythrocytosis and thrombocytopenia in congenital heart disease. Pediatr Res.

[147] Wong LL, Armugam A, Sepramaniam S, Karolina DS, Lim KY (2015). Circulating microRNAs in heart failure with reduced and preserved left ventricular ejection fraction. Eur J Heart Fail.

[148] Fichtlscherer S, Zeiher AM, Dimmeler S (2011). Circulating microRNAs: biomarkers or mediators of cardiovascular diseases?. Arterioscler Thromb Vasc Biol.

[149] Fukushima Y, Nakanishi M, Nonogi H, Goto Y, Iwai N (2011). Assessment of plasma miRNAs in congestive heart failure. Circ J.

[150] Wei XJ, Han M, Yang FY, Wei GC, Liang ZG (2015). Biological significance of miR-126 expression in atrial fibrillation and heart failure. Braz J Med Biol Res.

[151] Nandi SS, Duryee MJ, Shahshahan HR, Thiele GM, Anderson DR, Mishra PK (2015). Induction of autophagy markers is associated with attenuation of miR-133a in diabetic heart failure patients undergoing mechanical unloading. Am J Transl Res.

[152] Nair N, Kumar S, Gongora E, Gupta S (2013). Circulating miRNA as novel markers for diastolic dysfunction. Mol Cell Biochem.

[153] Miyamoto SD, Karimpour-Fard A, Peterson V, Auerbach SR, Stenmark KR (2015). Circulating microRNA as a biomarker for recovery in pediatric dilated cardiomyopathy. J Heart Lung Transplant.

[154] Zeng X, Li X, H W (2017). Expression of circulating microRNA-182, CITED2 and HIF-1 in ischemic cardiomyopathy and their correlation. J Clin Cardiol.

[155] Akat KM, Moore-McGriff DV, Morozov P, Brown M, Gogakos T (2014). Comparative RNA-sequencing analysis of myocardial and circulating small RNAs in human heart failure and their utility as biomarkers. Proc Natl Acad Sci U S A.

[156] Leger KJ, Singh S, Canseco D, vonGrote EC, Karim-Ud-Din S, Collins SC et al. Identification of Novel Circulating microRNAs in Ischemic Cardiomyopathy Utilizing Whole Blood microRNA Profiling. Am Heart Assoc; 2013.

[157] van Boven N, Kardys I, van Vark LC, Akkerhuis KM, de Ronde MWJ (2018). Serially measured circulating microRNAs and adverse clinical outcomes in patients with acute heart failure. Eur J Heart Fail.

